# Neochordae implantation versus leaflet resection in mitral valve posterior leaflet prolapse and dilated left ventricle: a propensity score matching comparison with long-term follow-up

**DOI:** 10.1093/ejcts/ezad274

**Published:** 2023-08-08

**Authors:** Benedetto Del Forno, Kevin Tavana, Claudio Ruffo, Davide Carino, Elisabetta Lapenna, Guido Ascione, Arturo Bisogno, Igor Belluschi, Maria Giovanna Scarale, Alessandro Nonis, Fabrizio Monaco, Ottavio Alfieri, Alessandro Castiglioni, Francesco Maisano, Michele De Bonis

**Affiliations:** Department of Cardiac Surgery, IRCCS San Raffaele Hospital, Vita-Salute San Raffaele University, Milan, Italy; Department of Cardiac Surgery, IRCCS San Raffaele Hospital, Vita-Salute San Raffaele University, Milan, Italy; Department of Cardiac Surgery, IRCCS San Raffaele Hospital, Vita-Salute San Raffaele University, Milan, Italy; Department of Cardiac Surgery, IRCCS San Raffaele Hospital, Vita-Salute San Raffaele University, Milan, Italy; Department of Cardiac Surgery, IRCCS San Raffaele Hospital, Vita-Salute San Raffaele University, Milan, Italy; Department of Cardiac Surgery, IRCCS San Raffaele Hospital, Vita-Salute San Raffaele University, Milan, Italy; Department of Cardiac Surgery, IRCCS San Raffaele Hospital, Vita-Salute San Raffaele University, Milan, Italy; Department of Cardiac Surgery, IRCCS San Raffaele Hospital, Vita-Salute San Raffaele University, Milan, Italy; University Centre of Statistics in Biomedical Sciences (CUSSB), Vita-Salute San Raffaele University, Milan, Italy; University Centre of Statistics in Biomedical Sciences (CUSSB), Vita-Salute San Raffaele University, Milan, Italy; Department of Anesthesiology, IRCCS San Raffaele Hospital, Vita-Salute San Raffaele University, Milan, Italy; Department of Cardiac Surgery, IRCCS San Raffaele Hospital, Vita-Salute San Raffaele University, Milan, Italy; Department of Cardiac Surgery, IRCCS San Raffaele Hospital, Vita-Salute San Raffaele University, Milan, Italy; Department of Cardiac Surgery, IRCCS San Raffaele Hospital, Vita-Salute San Raffaele University, Milan, Italy; Department of Cardiac Surgery, IRCCS San Raffaele Hospital, Vita-Salute San Raffaele University, Milan, Italy

**Keywords:** Mitral valve repair, Neochoardae implantation, Posterior leaflet resection, Dilated left ventricle, Reverse remodelling

## Abstract

**OBJECTIVES:**

Uncorrected severe mitral regurgitation (MR) due to posterior prolapse leads to left ventricular dilatation. At this stage, mitral valve repair becomes mandatory to avoid permanent myocardial injury. However, which technique among neochoardae implantation and leaflet resection provides the best results in this scenario remains unknown.

**METHODS:**

We selected 332 patients with left ventricular dilatation and severe degenerative MR due to posterior leaflet (PL) prolapse who underwent neochoardae implantation (85 patients) or PL resection (247 patients) at our institution between 2008 and 2020. A propensity score matching analysis was carried on to decrease the differences at baseline.

**RESULTS:**

Matching yielded 85 neochordae implantations and 85 PL resections. At 10 years, freedom from cardiac death and freedom from mitral valve reoperation were 92.6 ± 6.1% vs 97.8 ± 2.1% and 97.7 ± 2.2% vs 95 ± 3% in the neochordae group and in the PL resection group, respectively. The MR ≥2+ recurrence rate was 23.9 ± 10% in the neochordae group and 20.8 ± 5.8% in the PL resection group (*P* = 0.834) at 10 years. At the last follow-up, the neochordae group showed a higher reduction of left ventricular end-diastolic diameter (44 vs 48 mm; *P* = 0.001) and a better ejection fraction (60% vs 55%; *P* < 0.001) compared to PL resection group.

**CONCLUSIONS:**

In this subgroup of patients, both neochordae implantation and leaflet resection provide excellent durability of the repair in the long term. Neochordae implantation might have a better effect on dilated left ventricle.

**Figure:**
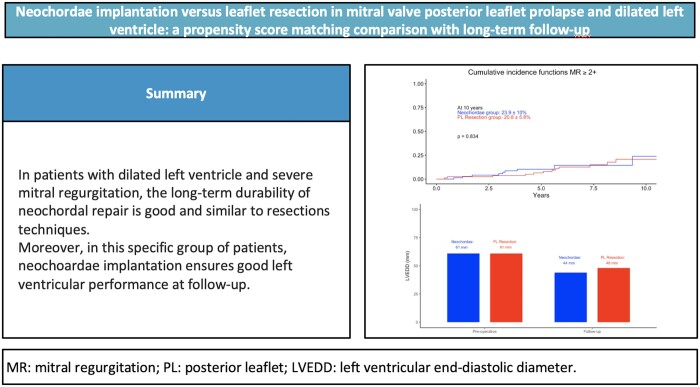

## INTRODUCTION

Surgical mitral valve (MV) repair represents the treatment of choice to address severe degenerative mitral regurgitation (MR). In this context, posterior leaflet (PL) prolapse is the most common lesion and it is usually treated by leaflet resection or neochordae implantation [1].

Resection techniques, either triangular or quadrangular, often associated with sliding or folding plasty have been introduced and popularized by Carpentier [2] in the early 80s. These techniques have been widely adopted and greatly stood the test of time [3, 4].

Conversely, based on the early work of Frater [5], David *et al.* [6, 7] started using PTFE sutures for neochordae replacement with excellent early and long-term results. More recently, Perier *et al.* [8] proposed the so-called ‘respect approach’ mainly based to avoid the removal of leaflet tissue and to implant PTFE neochorde to restore the physiological motion of the valve.

The comparison between these 2 repair strategies is still an object of ongoing debate, but no clear differences in results have been observed [9, 10].

Indeed, if not timely corrected, the persistent volume overload leads to left ventricular (LV) dilatation and remodelling [11]. Although LV reverse remodelling can occur after surgical correction of MR, it is unclear whether this process is related to the surgical technique and does have an impact on the long-term durability of the repair. In particular, it remains unknown the effect of significant LV reverse remodelling on implanted chordal length and its consequences on MR recurrence.

The aim of this study was to compare the early and long-term clinical and echocardiographic outcomes of resection techniques versus artificial chordae implantation, specifically in patients with PL prolapse and dilated left ventricle.

## PATIENTS AND METHODS

### Ethical statement

The Ethical Committee of the San Raffaele Hospital approved the study (115/INT/2022) and waived the individual informed consent for this retrospective anonymous analysis.

### Study population

From January 2008 to December 2020, 856 patients with LV dilatation and severe degenerative MR due to PL prolapse underwent MV repair at San Raffaele University Hospital, Milan, Italy.

We included in this study patients with LV dilatation defined as a left ventricular end-diastolic diameter (LVEDD) ≥58 mm in male and ≥53 mm in female, according to the position paper of the American Society of Echocardiography and the European Association of Cardiovascular Imaging [12].

For the purpose of the study and to minimize all possible confounding factors, we selected patients who underwent neochordae implantation or PL resection techniques, namely triangular resection or quadrangular resection associated with folding plasty. Therefore, we excluded 416 patients who were treated with different repair techniques such as quadrangular resection associated with sliding plasty or annular plication, central or commissural edge-to-edge, chordal transposition and ‘butterfly’ technique. Moreover, we excluded 108 patients with one of the following conditions: severe LV dysfunction, urgent or emergency operation, concomitant infective endocarditis, previous mediastinal radiation therapy or hypertrophic obstructive cardiomyopathy.

Finally, 332 patients were selected and represented the overall cohort of the study. Neochordae implantation was performed in 85 patients whereas PL resection in 247 patients.

In general practice, the surgeon’s preference played the major role in the choice of the reparative technique. To mitigate this selection bias and to obtain 2 balanced groups of patients, a propensity score matching was used. This methodology allowed us to achieve 2 similar groups (85 patients each), with respect to the preoperative characteristics, that have then been used for the analysis.

Preoperative, intraoperative and postoperative data were collected through our hospital database.

### Surgical techniques

The operations were carried out through both conventional median sternotomy or right-sided anterolateral minithoracotomy, with moderately hypothermic cardiopulmonary bypass and cold crystalloid cardioplegia.

The MV was exposed through a conventional left atrial incision, parallel to the interatrial groove. According to the inclusion criteria of the study, in the PL resection group, the technique of repair was a triangular resection of the central scallop of the PL (P2) (47 patients, 55%) or a limited quadrangular resection with folding plasty (38 patients, 45%). Conversely, in the neochordae group, artificial PTFE neochordae were implanted to address the P2 lesion. In our series, 50 patients (59%) underwent standard ‘hand-adjusted’ neochordae implantation (median number of neochordae implanted: 2) and 35 patients (41%) underwent premeasured loops technique (median number of loops implanted: 3; median loops length: 14 mm). In all patients, a prosthetic annuloplasty was associated.

Concomitant procedures, such as tricuspid valve repair, coronary artery bypass grafting and atrial fibrillation (AF) ablation, were associated whenever indicated.

### Statistical analyses

**Figure 1:**
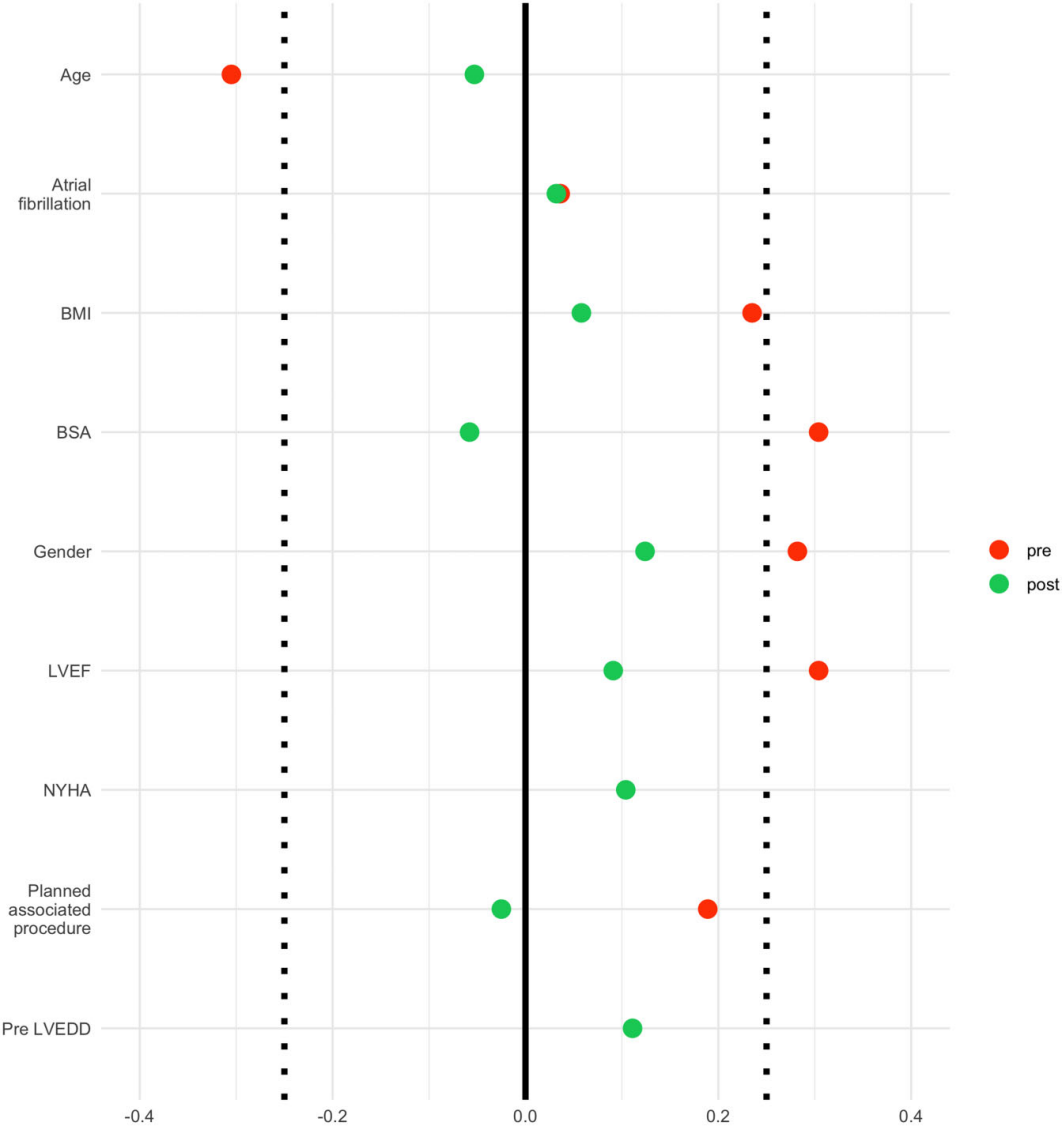
Love plot displaying covariate balance pre- and post-matching. Vertical dotted lines at ±0.25 indicate the acceptability bounds. After matching all variables stand within the acceptability threshold [13].

Propensity score matching was performed using exclusively all the complete variables such as sex, age, body surface area, hypertension, preoperative AF, New York Heart Association (NYHA) functional class, preoperative left ventricular ejection fraction (LVEF), preoperative LVEDD and planned associated procedures. The matching was used to randomly select the subgroup of patients undergoing PL resection to be compared to the group of patients undergoing neochordae implantation. Patients were weighted according to the propensity score and the samples were matched at 1:1 ratio without replacement. Standardized mean differences have been used to evaluate the quality of the matching (Fig. 1) [13].

Continuous variables were reported as median and interquartile range [IQR 25th percentile; 75th percentile], whereas categorical variables were reported as total frequencies and percentages. Two-sided *P*-values for continuous variables refer to Kruskal–Wallis test. Two-sided *P*-values for categorical variables refer to Fisher’s exact test when appropriate.

Kaplan–Meier method was used to estimate overall survival, freedom from cardiac death and freedom from MV reintervention for each group of intervention.

According to Peduzzi *et al.* [14], we decided to not compute inferential comparison between the neochordae group and the PL resection group for overall survival, freedom from cardiac death and freedom from MV reoperation because of the low number of events (<10) in each outcome.

The main outcome was MR recurrence ≥2. Competing risks proportional-hazards regression model, following the Fine–Gray model, for time to MR ≥2 with death as competing risk was performed. Cumulative incidence function (CIF) for time to MR ≥2 with death as competing risk was calculated.

Risks were reported as hazard ratios along with their 95% confidence intervals. A *P*-value of <0.05 was considered significant. All analyses were performed using R statistical software (version 4.0.4; https://cran.r-project.org/index.html). The R package MatchIt was used to implement propensity score matching. The R packages survival and cmprsk were used to perform survival and competing risk analyses.

### Echocardiographic evaluation

All patients underwent preoperative transoesophageal echocardiography focused to confirm the severity of the MR and to identify and better define the characteristic of PL lesions. In this cohort, all patients showed an isolated prolapse or flail of the central portion of the PL (P2). A transoesophageal echocardiography was routinely repeated immediately after weaning from cardiopulmonary bypass. A transthoracic echocardiography examination was performed in all patients before hospital discharge and was available in all patients alive and who were not lost at follow-up. To evaluate the recurrence of MR, an integrative approach was used to define MR severity. A non-linear four-grade scale was adopted to define MR as mild (1+/4+), mild to moderate (2+/4+), moderate to severe (3+/4+) and severe (4+/4+).

### Follow-up

Follow-up data were obtained by means of outpatient visit and transthoracic echocardiography performed in our Institution in 75% of the cases. In the remaining patients, the follow-up data were acquired with telephone interview with the patients and referring cardiologists. We focused on survival, causes of death, incidence of MV reoperation, recurrence of MR ≥2+, clinical status, symptoms and echocardiographic parameters. When the transthoracic echocardiography was performed in a different institution, the report was collected for review. Transthoracic echocardiography data regarding the degree of MR, LVEF and LVEDD were available for all the patients alive and who were not lost at follow-up. We conducted follow-up examinations in the same period for all patients, irrespective of the time since the operation occurred (common closing date method). The cause of death was determined from death certificates or from information from the physician who was caring for the patient at that time. Follow-up was 96% complete. The median clinical and echocardiographic follow-up time was 5.97 years [4.49–9.61] with a maximum follow-up time of 13.76 years.

## RESULTS

Among 332 overall patients, 85 (25.6%) underwent neochordae implantation and 247 (74.4%) underwent PL resection. Matching yielded 85 neochordae implantation and 85 PL resections. Matched groups were well balanced and there were no significant differences in both groups with regard to the preoperative clinical characteristics (Table 1). The median age was 63 years [IQR 52–70] in the neochordae group and 63 years [IQR 53–69.3] in the PL resection group (*P* = 0.875). Eleven patients (12.9%) in the neochordae group and 14 patients (16.5%) in the PL resection group were in NYHA functional class III or IV (*P* = 0.326). The median LVEF was 64% [IQR 60–68] in the neochordae group and 65% [IQR 60.0–69.0] in the PL resection group (*P* = 0.727) and the median LVEDD was 61 mm [IQR 57–63] in the neochordae group and 61 mm [IQR 59.0–64.0] in the PL resection group (*P* = 0.433).

**Table 1:** Preoperative features (unmatched and matched groups)

Variables	Neochordae group, unmatched (85 patients)	PL-resection group, unmatched (247 patients)	*P*-Value	SMD	Neochordae group, matched (85 patients)	PL-resection group, matched (85 patients)	*P*-value	SMD
Male sex, *n* (%)	53 (62.4)	186 (75.3)	0.031	0.282	53 (62.4)	58 (68.2)	0.519	0.124
Age (years), median (IQR)	63 [52–70]	57 [48–67]	0.021	−0.305	63 [52–70]	63 [53–69]	0.875	−0.053
BSA, median (IQR)	1.83 [1.72–1.92]	1.89 [1.78–2.02]	0.001	0.304	1.83 [1.72–1.92]	1.84 [1.70–1.94]	0.929	−0.058
Hypertension, *n* (%)	34 (40)	64 (25.9)	0.02		34 (40)	26 (30.6)	0.261	
NYHA class III/IV, *n* (%)	11 (12.9)	29 (11.7)	0.465	0.420	11 (12.9)	14 (16.5)	0.702	0.104
Atrial fibrillation, *n* (%)	14 (16.5)	44 (17.8)	0.908	0.036	14 (16.5)	13 (15.3)	1	0.032
Coronary artery disease, *n* (%)	9 (10.6)	21 (8.5)	0.143		9 (10.6)	9 (10.6)	1	
Tricuspid regurgitation, *n* (%)			0.03				0.669	
Moderate	25 (29.4)	43 (17.4)			25 (29.4)	20 (23.5)		
Severe	1 (1.2)	5 (2)			1 (1.2)	1 (1.2)		
Planned associated procedure, *n* (%)	29 (34.1)	63 (25.5)	0.165	0.189	29 (34.1)	28 (32.9)	1	−0.025
LVEF, median (IQR)	64.5 [60–68]	62 [59–67]	0.039	0.304	64 [60–68]	65 [60–69]	0.727	0.091
Pre-LVEDD, median (IQR)	61 [57–63]	63 [60–65]	<0.001	0.462	61 [57–63]	61 [59–64]	0.433	0.111
Pre-LVESD, median (IQR)	36 [30–40]	41 [36–46]	<0.001		36 [30–40]	38 [34–43]	0.101	
sPAP, median (IQR)	37 [34–43]	37 [30–46]	0.558		37 [34–43]	36 [30–50]	0.703	

BSA: body surface area; IQR: interquartile range; LVEDD: left ventricular end-diastolic diameter; LVEF: left ventricular ejection fraction; LVESD: left ventricular end-systolic diameter; NYHA: New York Heart Association; PL: posterior leaflet; SMD: standardized mean differences; sPAP: systolic pulmonary artery pressure.

### In-hospital outcomes

Operative characteristics of the matched population are shown in Table 2. Only 1 in-hospital death (1.2%) occurred in neochordae group whereas no patient died in the PL resection group. Right minithoracotomy approach was performed in 21 patients (24.7%) of the neochordae group and in 15 patients (17.6%) of the PL resection group (*P* = 0.348). The median ring size was 35 mm [IQR 33–36] in the neochordae group and 35 mm [IQR 33–35] in the PL resection group (*P* = 0.954). CPB time was 82 min [IQR 68–100] in the neochordae group and 74 min [IQR 66–90] in the PL resection group (*P* = 0.013) whereas aortic cross-clamp time was 62 min [IQR 52–78] in the neochordae group and 55 min [IQR 48–68] in the PL resection group (*P* = 0.019). Four patients (4.7%) in the neochordae group and 3 patients (3.5%) in the PL resection group needed intra-aortic balloon pump (*P* = 1.0). Sixteen patients (19.0%) in the neochordae group and 19 patients (22.9%) in the PL resection group developed AF during postoperative hospitalization (*P* = 0.674). Echocardiography performed at hospital discharge showed residual MR 2+ in 2 patients (2.4%) in the neochordae group and in 5 patients (6.2%) in the PL resection group (*P* = 0.623). All other patients of both groups presented mild (1+) or no residual postoperative MR.

**Table 2:** Operative and postoperative data (matched groups)

Variables	Neochordae group, 85 patients	PL resection group, 85 patients	*P*-Value
Ring size (mm), median (IQR)	35 [33–36]	35 [33–35]	0.954
Right minithoracotomy, *n* (%)	21 (24.7%)	15 (17.6%)	0.348
CPB time (min), median (IQR)	82 [68–100]	74 [66–90]	0.013
Aortic cross-clamp time (min), median (IQR)	62 [52–78]	55 [48–68]	0.019
Coronary artery bypass grafting, *n* (%)	7 (8.2)	9 (10.6)	0.433
Tricuspid valve repair, *n* (%)	21 (24.7)	16 (19)	0.479
Atrial fibrillation ablation, *n* (%)	10 (11.7)	6 (7)	0.37
IABP, *n* (%)	4 (4.7)	3 (3.5)	1
Postoperative atrial fibrillation, *n* (%)	16 (19.0)	19 (22.9)	0.674
MR at discharge			0.623
Grade 0, *n* (%)	42 (49.4)	43 (50.6)	
Grade 1, *n* (%)	41 (48.2)	37 (43.5)	
Grade 2, *n* (%)	2 (2.3)	5 (6.2)	
Hospital stay, median (IQR)	5 [4–6]	5 [4–7]	0.528
Death, *n* (%)	1 (1.2)	0	

CBP: cardiopulmonary bypass; IABP: intra-aortic balloon pump; IQR: interquartile range; MR: mitral regurgitation; PL: posterior leaflet.

**Table 3:** Predictors of mitral regurgitation recurrence ≥2+ (Fine–Gray model)

	Univariable			Multivariable		
	HR	*P*-Value	95% CI	HR	*P*-Value	95% CI
Matched groups comparison	0.91	0.834	0.39–2.13			
Age	1.02	0.068	1.00–1.03	1.01	0.223	0.99–1.03
LVEF	0.97	0.215	0.93–1.02			
Female sex	0.63	0.254	0.28–1.4			
IABP	1.64	0.493	0.40–6.79			
Planned associated procedures	1.38	0.441	0.61–3.15			
sPAP	1.0	0.963	0.97–1.03			
AF	1.69	0.297	0.63–4.53			
MR at discharge ≥1	2.15	0.070	0.94–4.93	2.71	0.030	1.10–6.67
NYHA class ≥2	3.76	0.031	1.13–12.56	3.47	0.064	0.99–1.03

AF: atrial fibrillation; CI: confidence interval; HR: hazard ratio; IABP: intra-aortic balloon pump; LVEF: left ventricular ejection fraction; MR: mitral regurgitation; NYHA: New York Heart Association; sPAP: systolic pulmonary artery pressure.

### Follow-up outcomes

**Figure 2:**
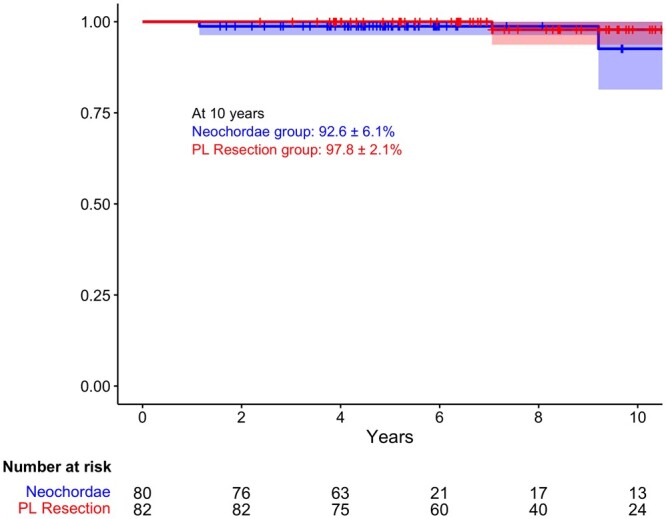
Ten-year Kaplan–Meier freedom from cardiac death for both groups.

During follow-up of the matched population, 7 patients (4%) in the neochordae group and 2 patients (1%) in the PL resection group died. Five deaths (3%) were cardiac related: 4 in the neochordae group and 1 in the PL resection group. The 10-year overall survival was 84.6 ± 7.6% in the neochordae group and 96.6 ± 2.4% in the PL resection group. At 10 years, the freedom from cardiac death was 92.6 ± 6.1% in the neochordae group and 97.8 ± 2.1% in the PL resection group (Fig. 2).

**Figure 3:**
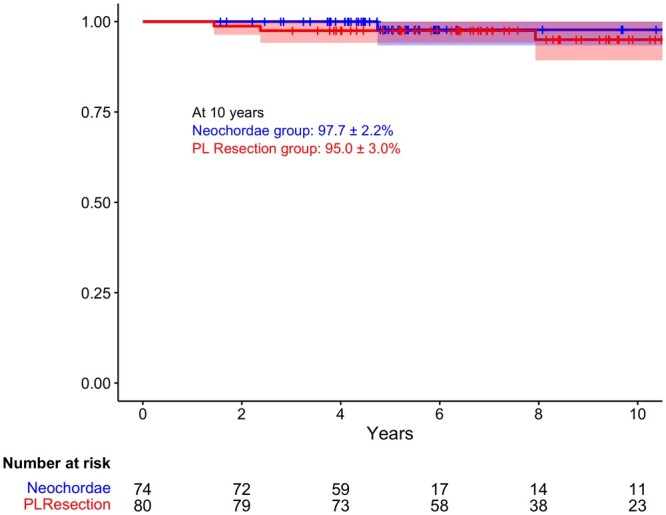
Ten-year Kaplan–Meier freedom from mitral valve reoperation for both groups.

Moreover, 5 patients required reoperation: 2 patients (1%) of the neochordae group and 3 patients (2%) of the PL resection group. The freedom from MV reoperation, was 97.7 ± 2.2% in the neochordae group and 95 ± 3% in the PL resection group at 10 years (Fig. 3).

**Figure 4:**
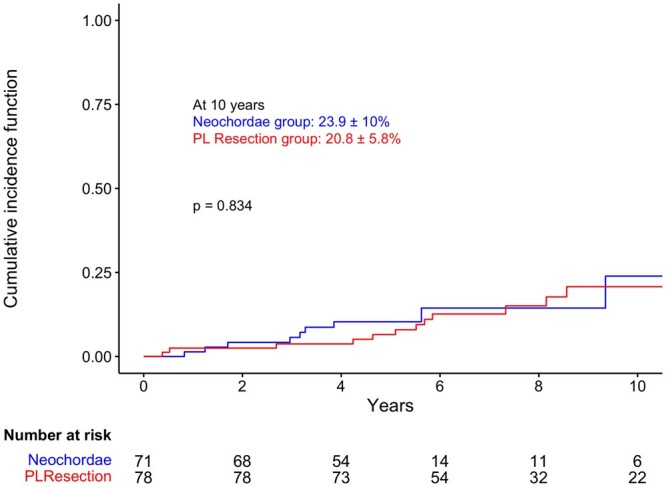
Cumulative incidence function of mitral regurgitation recurrence ≥2+ with death as competing risk in both groups.

The CIF of MR recurrence ≥2+, with death as competing risk, was 23.9 ± 10% in the neochordae group and 20.8 ± 5.8% in the PL resection group (*P* = 0.834) at 10 years (Fig. 4). Specifically, 9 patients in the neochordae group and 15 patients in the PL resection group had this event. MR ≥1+ at discharge (hazard ratio 2.71, 95% confidence interval [1.10–6.67], *P* = 0.030) was the only predictor of MR recurrence ≥2+ in the long term (Table 3). At the last follow-up, only 1 patient (1.3%) in the neochordae group and 3 patients (3.7%) in the PL resection group had MR 3+.

At the last follow-up, 11 patients (14.1%) in the neochordae group and 21 patients (31%) in the PL resection group were affected by AF (*P* = 0.032). The median LVEDD was 44 mm [IQR 42–47] in the neochordae group and 48 mm [IQR 43.7–50] in the PL resection group (*P* = 0.001).

In the neochordae group, 41 patients (54.7%) were in NYHA class I, 33 patients (44%) were NYHA class II and 1 patient (1.3%) was in NYHA class III whereas in the PL resection group, 25 patients (35.7%) were in NYHA class I, 43 patients (61.4%) were NYHA class II and 2 patients (2.9%) were in NYHA class III (*P* = 0.05). The median EF was 60% [IQR 58–63] in the neochordae group and 55% [IQR 55–60] in the PL resection group (*P* < 0.001).

## DISCUSSION

The findings of this retrospective propensity score matching analysis showed that neochordae implantation and PL resection provide similar excellent results in terms of survival and durability of the repair in patients with severe MR and dilated left ventricle.

This kind of patients represents a specific subgroup in whom the continuous LV volume overload due to the uncorrected chronic MR results in a progressive overstretching of cardiomyocytes and consequent enlargement of the left ventricle. At this stage, MV repair becomes mandatory as well as the choice of the most appropriate reparative technique. The aim of surgery is both to interrupt the vicious cycle before irreversible myocardial damage occurs and to promote LV reverse remodelling.

After MV repair, LV reverse remodelling happens in 2 distinct phases. In the first stage, there is a decrease in LV end-diastolic volume with no change in LV end-systolic volume, leading to a decrease in LVEF. The second stage of reverse remodelling is characterized by an improvement in systolic function, due to the decrease in LV end-systolic volume and consequent improvement of LVEF [15].

In the context of PL pathology, PL resection and neochordae implantation have been the most adopted techniques over the last 4 decades.

Generally speaking, MV repair aims to restore physiological leaflet motion, create a sufficient surface of coaptation with adequate orifice area and stabilize the mitral annulus. Although resection techniques have provided durable and haemodynamically satisfactory results with excellent freedom from reoperation, when performed in dedicated centres, partial resection of the PL alters its geometry and its physiological motion. Moreover, when those techniques are associated with annular plication and sliding plasty, the crimping of the posterior annulus can lead to a detrimental effect on LV performance [6].

On the other hand, neochordae implantation follows the main principles of reparative MV surgery providing largest orifice area and surface of coaptation and better preservation of the ventriculo-annular continuity.

Based on this hypothesis, neochordae implantation might be the most appropriate technique, especially in patient with altered LV geometry. However, it remains unknown if the subsequent LV reverse remodelling after MR correction does influence the length of the implanted neochoardae potentially resulting in recurrent PL prolapse. Neochordal repair can be performed by either using ‘hand-adjusted’ PTFE neochordae or premeasured loops. It is noteworthy that in our series both these techniques have been used.

With respect to cardiac death and rate of MV reoperation, both groups showed excellent results although we cannot provide inferential comparison given the low number of events. Regarding MR recurrence, remarkably only 1 patient in the neochordae group and 3 patients in PL resection group had MR 3+ at the last follow-up. At 10 years, the CIF of MR recurrence ≥2+, with death as competing risk, was similar between the 2 groups. The Fine and Gray confirmed that achieving an immediate optimal result is the best predictor of the durability of the repair.

The comparison of reoperation rate has been objected to by several studies. Pfannmueller *et al.* recently reported their results in a large cohort of patients undergoing minimally invasive MV repair with neochordae implantation by using premeasured loops or resection techniques. At 10 years, they showed an excellent freedom from MV reoperation in both group (total 96.7%) without statistically significant difference between the 2 groups [16]. Lange *et al.* showed similar freedom from MV-related reoperation between patients undergoing standard ‘hand-adjusted’ chordal replacement or PL resection at 4 years. At the last follow-up, they observed that 94% of patients had no or mild MR without difference between the 2 groups [10].

Another finding of our research is that a significant reduction in LVEDD was observed in both groups. This occurrence did not impact the performance of the nechordae implantation repair, thus confirming that this technique can be adopted even in patients with dilated left ventricles.

Moreover, in our series, the neochordae group showed a better LVEF and a smaller LVEDD as compared to the resection group at the latest follow-up. In a large meta-analysis, Mazine *et al.* [17] also reported higher postoperative LVEF in patients undergoing chordal replacement technique. This conclusion is mainly based on the results of the research by Imasaka *et al.*, who analysed the haemodynamic performance of 72 patients who underwent neochoardae implantation or PL resection. One month after surgery, they observed a better improvement of LVEF in the neochoardae group. The researchers theorized that preservation of the ventriculo-annular continuity could be a possible explanation for their findings [18]. Conversely, van Wijngaarden *et al.* [19] investigated the LV function using LV global longitudinal strain in patients undergoing chordal replacement or PL resection. They reported similar LV performances both at post-operative evaluation and at 2-year follow-up.

With respect to the evolution of the LVEDD, a recent sub-analysis of the CAMRA Trial showed no differences in terms of reduction of the LVEDD and LV end-diastolic volume at 12-month follow-up. The authors concluded that the MV repair techniques did not influence the postoperative LV reverse remodelling [20].

Our research differs from previous ones in 2 main aspects. First of all, we analysed exclusively patients with dilated left ventricle. Second, we observed these patients at longer follow-up. In our opinion, these 2 points could explain our different results. Probably, in patients with dilated left ventricle, the benefit due to the better preservation of the ventriculo-annular continuity are more evident.

### Limitations

First of all, this is a retrospective single-centre report and therefore subject to the inherent weaknesses of a retrospective analysis. Second, to define the left ventricle dilatation, we could just use the LVEDD and not the LV end-diastolic volume which was not available in all patients. Thirdly, we used a common closing date method to acquire follow-up data. This methodology may have generated differences in the follow-up period between the 2 groups, which could have an impact on the results. Fourth, in Kaplan–Meier analysis for overall survival, freedom from cardiac death and freedom from MV reoperation, we were not able to provide an inferential comparison between the 2 groups given the low numbers of events. Finally, after the first echocardiogram (within 30 days), only 75% of the patients had their exams performed at our institutions, while the remaining patients were followed by the referring cardiologist and therefore, we could just acquire the echocardiogram reports.

## CONCLUSION

In patients with dilated left ventricle and severe MR due to PL prolapse, both leaflet resection and neochordae implantation provide excellent long-term results in terms of survival and durability of the repair.

Neochoardae implantation might result in a higher reduction of LVEDD and a better LVEF as compared to leaflet resection. In our series, the reduction of LVEDD after chordal implantation does not lead to higher recurrence of PL prolapse and MR.

## Data Availability

The datasets analysed in the current study are available from the corresponding author on reasonable request.

## Notes

Presented at the 36th EACTS Annual Meeting, Milan, Italy, 5–8 October 2022.

## ABBREVIATIONS

AF: Atrial fibrillation
CIF: Cumulative incidence function
IQR: Interquartile range
LV: Left ventricular
LVEF: Left ventricular ejection fraction
LVEDD: Left ventricular end-diastolic diameter
MR: Mitral regurgitation
MV: Mitral valve
NYHA: New York Heart Association
PL: Posterior leaflet
